# AttenResNet18: A Novel Cross-Domain Fault Diagnosis Model for Rolling Bearings

**DOI:** 10.3390/s25195958

**Published:** 2025-09-24

**Authors:** Gangjin Huang, Shanshan Wu, Yingxiao Zhang, Wuguo Wei, Weigang Fu, Junjie Zhang, Yuxuan Yang, Junheng Fu

**Affiliations:** 1 College of Aviation Engineering, Civil Aviation Flight University of China, Guanghan 618307, China; huanggj@cafuc.edu.cn (G.H.); weiwuguo@163.com (W.W.); jiaodafwg@126.com (W.F.); junjiezhang2018@163.com (J.Z.); yyx@cafuc.edu.cn (Y.Y.); 2 Sichuan Provincial Engineering Research Center for Flight Technology and Flight Safety, Civil Aviation Flight University of China, Guanghan 618300, China; 3 College of Information Engineering, Sichuan Agricultural University, Ya’an 625000, China; 202205821@stu.sicau.edu.cn (S.W.); 202205721@stu.sicau.edu.cn (Y.Z.); 4 College of Water Conservancy and Hydropower Engineering, Sichuan Agricultural University, Ya’an 625014, China

**Keywords:** fault transfer diagnosis, deep domain adaptation, self-attention mechanism, noisy environment, rolling bearing

## Abstract

To tackle the difficulties in cross-domain fault diagnosis for rolling bearings, researchers have devised numerous domain adaptation strategies to align feature distributions across varied domains. Nevertheless, current approaches tend to be vulnerable to noise disruptions and often neglect the distinctions between marginal and conditional distributions during feature transfer. To resolve these shortcomings, this study presents an innovative fault diagnosis technique for cross-domain applications, leveraging the Attention-Enhanced Residual Network (AttenResNet18). This approach utilizes a one-dimensional attention mechanism to dynamically assign importance to each position within the input sequence, thereby capturing long-range dependencies and essential features, which reduces vulnerability to noise and enhances feature representation. Furthermore, we propose a Dynamic Balance Distribution Adaptation (DBDA) mechanism, which develops an MMD-CORAL Fusion Metric (MCFM) by combining CORrelation ALignment (CORAL) with Maximum Mean Discrepancy (MMD). Moreover, an adaptive factor is employed to dynamically regulate the balance between marginal and conditional distributions, improving adaptability to new and untested tasks. Experimental validation demonstrates that AttenResNet18 achieves an average accuracy of 99.89% on two rolling bearing datasets, representing a significant improvement in fault detection precision over existing methods.

## 1. Introduction

In industrial machinery such as motors and turbines, rolling bearings are vital components, as their operational effectiveness significantly influences the dependability and stability of these systems [1,2,3]. When exposed to continuous high-speed conditions, these bearings become susceptible to localized defects, leading to heightened friction, increased energy dissipation, and potential risks such as unexpected shutdowns or safety hazards [4]. Therefore, implementing effective fault detection and diagnosis procedures for rolling bearings is essential for early defect identification, preventing fault progression, and facilitating predictive maintenance strategies, which collectively ensure the secure and efficient functioning of industrial equipment [5].

Traditional approaches to diagnosing faults in rolling bearings are typically categorized into three distinct groups. The first category is based on signal decomposition combined with envelope spectrum analysis. For instance, Li et al. [6] proposed a method that optimizes the influence parameters of Variational Mode Decomposition (VMD) based on the kurtosis of the envelope signal, which effectively reduces the workload. However, these methods rely heavily on expert experience for rule-based reasoning, making them inherently qualitative. When fault features are not identifiable, the diagnostic results are highly susceptible to subjectivity. The second category encompasses machine learning-based diagnostic methods. For instance, Wang et al. [7] combine Recurrence Quantification Analysis with a Support Vector Machine enhanced through Bayesian optimization (RQA–Bayes–SVM) for fault diagnosis. These methods allow for quantitative analysis but assume that the equipment operates under constant conditions. However, in practical industrial environments, equipment types, loads, and speeds often vary due to environmental changes, which limits the generalizability of the model. The third method leverages deep learning models to automatically derive features from vibration data, thus removing the reliance on manual feature extraction [8]. For instance, Dai et al. [9] apply one-dimensional Convolutional Neural Networks (CNNs) to evaluate the fused characteristics of vibration and acoustic signals for diagnosing bearing faults. Gao et al. [10] enhanced the diagnostic capability of a Deep Belief Network (DBN) by optimizing its performance through the application of the Salp Swarm Algorithm (SSA). Cui et al. [11] introduced a Feature Distance Stacked Autoencoder (FD-SAE) to accelerate training, following initial data categorization using a basic linear Support Vector Machine (SVM). However, these approaches typically presume that training and testing datasets exhibit identical distributions and demand extensive data volumes for training to guarantee precise outcomes.

Deep transfer learning [12,13,14] tackles the aforementioned challenges by providing an innovative approach to diagnosing bearing faults across diverse devices and operating conditions, achieved through cross-domain knowledge transfer. This method overcomes the traditional reliance on the assumption of identical distributions in diagnostic models, enabling adaptive diagnosis under complex conditions through an end-to-end feature learning mechanism. Among these methods, domain-adaptation-based transfer learning stands out by providing effective solutions for fault diagnosis under complex conditions by reducing discrepancies between domains and capturing consistent features.

In the field of rolling bearing fault diagnosis, domain adaptation methods are typically categorized into two paradigms: hybrid approaches that integrate physical modeling or simulation with machine learning, and data-driven domain adaptation methods. Hybrid methods leverage physical simulations to generate synthetic data, addressing challenges such as data scarcity in real-world scenarios, and combine these with machine learning to enhance fault classification performance. Such methods incorporate domain-specific physical knowledge, thereby improving model interpretability. For instance, Sobie et al. [15] utilized simulation models to provide training data for machine learning, achieving an accuracy of up to 94% across four experimental datasets. Matania et al. [16] fused physics-based algorithms with machine learning to propose a novel hybrid algorithm, enabling spall type classification through zero-fault learning to mitigate the issue of insufficient fault data. Ma et al. [17] constructed a dynamic simulation model for bearing faults to generate simulated signals, supplementing missing fault data, and combined these with measured signals to form relatively complete training datasets for fault diagnosis. However, these methods rely on precise physical models, and any errors in the simulations may propagate to the learning process.

Data-driven models can adapt to complex patterns without explicit physical prior knowledge and are primarily divided into adversarial training and statistical criteria-based approaches. Adversarial training-based methods, inspired by Generative Adversarial Networks (GANs) [18], align features through adversarial mechanisms. The Domain-Adversarial Neural Network (DANN), proposed by Ganin et al. [19], learns domain-invariant features via adversarial training, offering a novel approach for unsupervised domain adaptation. Building on this, Jin et al. [20] introduced the multi-channel and multi-scale CNN-LSTM-ECA (MMCLE) feature extraction module to effectively address domain shift issues. Long et al. [21] proposed the Conditional Domain Adversarial Network (CDAN), an extension of the DANN framework that conditions the domain discriminator on both feature representations and classifier predictions via multilinear transformations. However, adversarial training, which relies on the adversarial mechanism between the feature extractor and the domain discriminator, suffers from issues such as vanishing gradients and mode collapse, leading to unstable model training [22].

On the other hand, methods based on statistical distribution metrics achieve cross-domain feature transfer by minimizing discrepancies in inter-domain probability distributions. Fang et al. [23] employed a Deep Residual Shrinkage Network (DRSN) for feature extraction and combined MMD with Local Maximum Mean Discrepancy (LMMD) to align both marginal and conditional distributions, significantly enhancing diagnostic performance under varying conditions. Wang et al. [24] designed a transfer learning framework that leverages the CORAL alignment loss. Xu et al. [25] presented a cooperative diagnostic technique for analyzing vibration signals from rolling bearings, leveraging an advanced adaptive filtering (AF) method alongside joint distribution adaptation (JDA). In the work of Qian et al. [26], a novel approach named the Deep Discriminative Transfer Learning Network (DDTLN) was developed by integrating the improved Softmax (I-Softmax) loss function and an optimized improved joint distribution adaptation (IJDA) strategy, significantly enhancing diagnostic accuracy across different machines. Chen et al. [27] introduced the Joint Attention Adversarial Domain Adaptation (JAADA) approach, employing MMD to address differences in feature distributions and embedding both local and global attention mechanisms within a cohesive adversarial domain adaptation framework, thereby minimizing the effects of domain shift.

Despite significant progress in domain adaptation (DA), two key challenges remain. First, considerable bottlenecks persist in practical industrial applications. After they pass through complex mechanical transmission paths to sensors, vibration signals are prone to random noise and other disturbances, which obscure critical information and often lead to misdiagnosis. Second, concerning distribution alignment strategies, Wang et al. [28] presented a theoretical study that emphasizes the significant differences in the roles of marginal and conditional distributions throughout the feature transfer process. However, existing JDA methods typically assume equal significance for both. While the balanced distribution adaptation (BDA) method proposed by Gu et al. [29] can adaptively adjust the importance of marginal and conditional distributions in cross-domain scenarios, the weights of these distributions need to be determined via cross-validation, which incurs high computational costs in multi-task and large-scale data settings. Therefore, there is still a lack of an efficient dynamic weighting approach that can adapt to different diagnostic tasks.

To address the aforementioned challenges, this paper introduces the AttenResNet18 model designed for cross-domain fault diagnosis in rolling bearings. By incorporating a one-dimensional self-attention mechanism [30], the method effectively captures long-range dependencies and extracts crucial features, thereby reducing the impact of noise. Moreover, by integrating the CORAL and MMD techniques, a hybrid metric named MCFM is developed, which reduces distribution discrepancies. We also design a loss function to improve diagnostic accuracy further. This paper offers the following key contributions:

- A one-dimensional self-attention mechanism is integrated into the AttenResNet18 structure, effectively tackling the issue of noise obscuring fault-related features.
- A Dynamic Balance Distribution Adaptation (DBDA) mechanism is proposed, which constructs the MCFM by fusing the MMD and CORAL to reduce distribution discrepancies. Furthermore, an adaptive factor is introduced to automatically assign weights to the marginal and conditional distributions based on their relative importance, eliminating the need for additional cross-validation and thus enabling more efficient adaptation to different tasks.

This paper is organized as follows: Section 2 comprehensively describes the proposed method. Section 3 outlines the experimental validation and performance comparisons. Finally, Section 4 concludes the study and proposes directions for future research.

## 2. Proposed Method

### 2.1. Process of the Proposed Method

**Figure 1:**
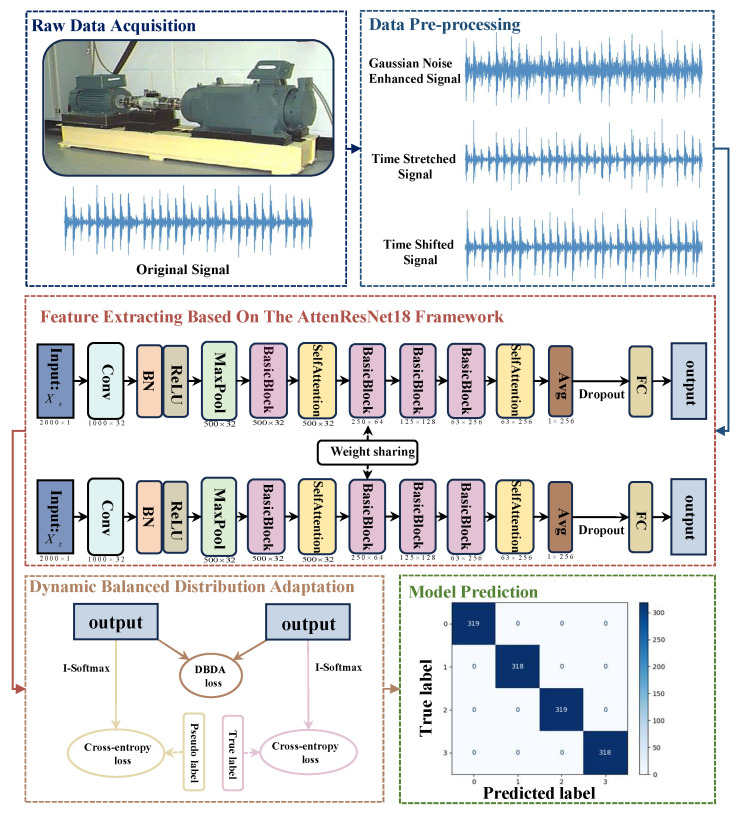
AttenResNet18 model fault diagnosis process.

The proposed approach’s workflow, shown in Figure 1, incorporates data preparation, feature acquisition, alignment, and fault identification.

### 2.2. Assumptions

- Distribution Shift: Both the marginal distributions $P(X_{S})\neq P(X_{T})$ and conditional distributions $P(Y_{S}|X_{S})\neq P(Y_{T}|X_{T})$ exhibit domain mismatch.
- Shared Label Space: $Y_{S}=Y_{T}$.

### 2.3. Data Preprocessing

This paper employs raw vibration signals as network inputs to avoid manual feature extraction, thereby reducing reliance on expert knowledge. The data processing workflow is as follows:

1. **Sliding Window Sampling:** A sliding window strategy is adopted to mitigate sample scarcity, generating continuous subsequences that preserve temporal characteristics and improve data utilization.
2. **Dataset Splitting:** To prevent data leakage, stratified sampling is directly applied to the original target domain dataset, dividing it into training and testing subsets with a 7:3 ratio. Although this strategy may introduce class imbalance in the test set, it better reflects the actual data distribution.
3. **Data Balancing:** Resampling techniques ensure a balanced class distribution in the training set.
4. **Data Augmentation and Standardization:** To enhance the model’s resilience against environmental disturbances and fluctuating operational states, specific data augmentation techniques are applied, followed by standardization. Table 1 details the augmentation methods.

### 2.4. Feature Extraction Based on the AttenResNet18 Framework

**Figure 2:**
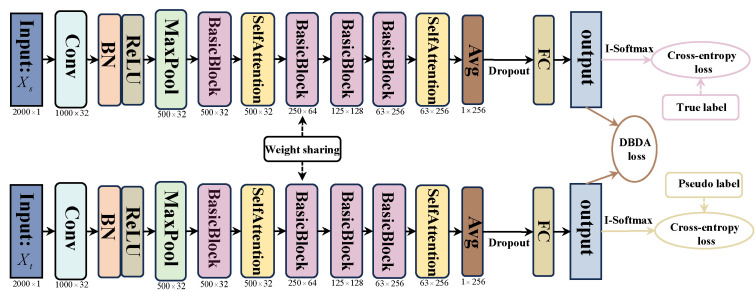
AttenResNet18 structure.

Affected by noise in the working environments and varying operating conditions, rolling bearings exhibit problems such as significant differences in feature distribution and fault features being easily masked by noise. In order to overcome these difficulties, we propose an innovative fault diagnosis method using AttenResNet18, as illustrated in Figure 2. By integrating a one-dimensional self-attention mechanism with transfer learning, this method enhances the adaptability of the model under varying working conditions.

**Figure 3:**
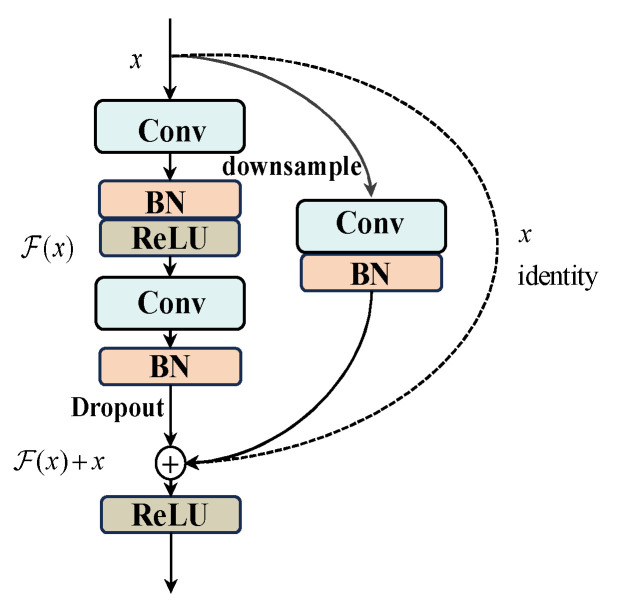
The BasicBlock structure.

Figure 3 shows the BasicBlock structure in AttenResNet18. The dotted shortcut branch is utilized when the input and output dimensions are identical, directly performing an identity mapping. The solid shortcut branch is employed when the input and output dimensions differ, achieving dimension matching through a convolutional layer.

**Figure 4:**
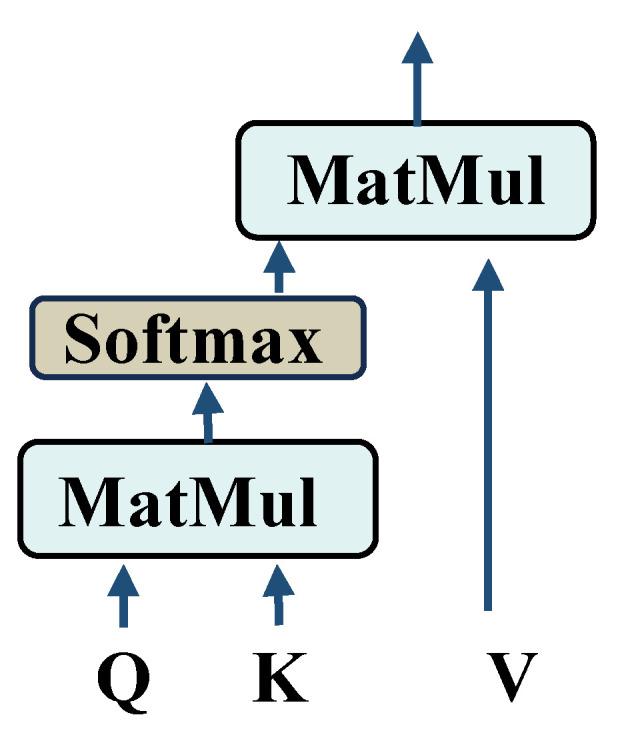
The one-dimensional self-attention mechanism structure.

Table 2 outlines the specific parameters of the AttenResNet18 model. This research used the classical ResNet18 [34] as the core architecture and introduced several enhancements. Initially, given the considerable complexity of the original ResNet18 framework, it tends to overfit to noise and extraneous details when analyzing bearing vibration signals affected by interference, potentially compromising its precision. To counteract this, we halved the channel count in each convolutional layer, thereby reducing both the parameter volume and computational load, which helps prevent overfitting to noise and non-pertinent characteristics. Dropout layers were incorporated within the BasicBlock and before the fully connected layer. Randomly dropping redundant computational units enhances the model’s regularization capability, mitigates overfitting, and improves both robustness to noise and generalization performance. Second, to overcome the locality limitation of traditional convolution, the one-dimensional self-attention mechanism was applied after extracting initial local features and higher-level abstract features, with its architecture illustrated in Figure 4.

Through adaptive weighting of each element in the input sequence, the one-dimensional self-attention mechanism facilitates the identification of significant features and the capture of distant dependencies. This enables it, at the lower level, to effectively filter and enhance local valuable information for fault diagnosis while suppressing noise, and, at the higher level, to learn global correlations among different features, thereby comprehensively improving feature representation capability.

### 2.5. Dynamic Balanced Distribution Adaptation

First, in situations involving unsupervised domain adaptation (UDA), particularly when precise labels are unavailable for the target domain, we adopt Bayes’ theorem, shown in Equation (1), to approximate the conditional probability distribution.

(1) $$P(Y=c|X)=\frac{P(X|Y=c)\cdot P(Y=c)}{P(X)}$$

Since P(X) is identical for all categories, we need only consider the numerator.

(2) P(Y=c|X)∝P(X|Y=c)⋅P(Y=c)

where P(X|Y=c) is computed as described in [35], and the prior probability of the class, P(Y=c), is determined by the ratio of class c instances.

(3) $$\left\{\begin{array}{l} P(Y_{S}=c)=\frac{N_{S}^{\left(c\right)}}{N_{S}}\\ P(Y_{T}=c)=\frac{N_{T}^{\left(c\right)}}{N_{T}} \end{array}\right.$$

where $N_{S}^{\left(c\right)}$ and $N_{T}^{\left(c\right)}$ represent the number of class c samples in the source and target domains, respectively, while $N_{S}$ and $N_{T}$ indicate the total samples in each domain.

In this research, pseudo-labels for target domain samples are produced using a classifier trained on source domain data. The conditional distribution alignment (CDA) is then achieved by repeatedly refining the estimates of class-conditional and prior probabilities in the target domain through an optimization process. The CDA mechanism can be formulated as follows:

(4) $$L_{\mathrm{CDA}}=\sum_{c=1}^{C}{\left\|E_{P(X_{S}|Y_{S}=c)}[T(X_{S})|Y_{S}=c]P(Y_{S}=c)-E_{P(X_{T}|Y_{T}=c)}[T(X_{T})|Y_{T}=c]P(Y_{T}=c)\right\|}^{2}$$

To reduce the discrepancy in marginal distributions, we further introduce the marginal distribution alignment (MDA) mechanism:

(5) $$L_{\mathrm{MDA}}={\left\|E_{P(X_{S})}[T(X_{S})]-E_{P(X_{T})}[T(X_{T})]\right\|}^{2}$$

By integrating the CDA and MDA mechanisms, the DBDA mechanism is formulated as follows:

(6) $$L_{\mathrm{DBDA}}=L_{\mathrm{CDA}}+L_{\mathrm{MDA}}$$

Furthermore, to address the complex and diverse nature of industrial operational environments, this study introduces a novel measure, named MCFM, which integrates the MMD, as defined within the Reproducing Kernel Hilbert Space (RKHS), with the CORAL method. By combining the domain discrepancy assessment techniques from MMD and CORAL, MCFM effectively reduces the distributional divergence between the source and target domains, thereby enhancing the model’s ability to generalize to various domains [36].

(7) $$D_{\mathrm{MCFM}}(X_{S},X_{T})=D_{\mathrm{CORAL}}(X_{S},X_{T})+D_{\mathrm{MMD}}(X_{S},X_{T})$$

where $D_{\mathrm{CORAL}}$ calculates the degree of discrepancy of second-order statistics between domains through the difference in covariance matrices:

(8) $$D_{\mathrm{CORAL}}=\frac{1}{4d^{2}}\|C_{S}-C_{T}{\|}_{F}^{2}$$

where d is the features’ dimensionality and $\|\cdot {\|}_{F}$ stands for the Frobenius norm. Additionally, $C_{S}$ and $C_{T}$ are the covariance matrices for the features in the source and target domains, respectively.

(9) $$\left\{\begin{array}{c} C_{S}=\frac{1}{N_{S}-1}\left(D_{S}^{⊤}D_{S}-\frac{1}{N_{S}}{\left(1^{⊤}D_{S}\right)}^{⊤}(1^{⊤}D_{S})\right)\\ C_{T}=\frac{1}{N_{T}-1}\left(D_{T}^{⊤}D_{T}-\frac{1}{N_{T}}{\left(1^{⊤}D_{T}\right)}^{⊤}(1^{⊤}D_{T})\right) \end{array}\right.$$

where 1 represents a column vector where each element is one, employed to calculate the average of the features.

$D_{\mathrm{MMD}}$ measures domain discrepancy using RKHS, as defined below:

(10) $$D_{\mathrm{MMD}}={\left\|\frac{1}{N_{S}}\sum_{i=1}^{N_{S}}\varphi (x_{S_{i}})-\frac{1}{N_{T}}\sum_{j=1}^{N_{T}}\varphi (x_{T_{j}})\right\|}_{H}^{2}$$

where H indicates the relevant RKHS, and φ(⋅) denotes the feature map to the RKHS; features are mapped using a Gaussian kernel-based function.

Incorporating the MCFM metric within the DBDA mechanism, the CDA loss function can be rewritten as

(11) $$L_{\mathrm{CDA}}=\sum_{c=1}^{C}D_{\mathrm{MCFM}}[(T(X_{S})|Y_{S}=c)\cdot P(Y_{S}=c),(T(X_{T})|Y_{T}=c)\cdot P(Y_{T}=c)]$$

The MDA loss function can be rewritten as

(12) $$L_{\mathrm{MDA}}=D_{\mathrm{MCFM}}(T(X_{S}),T(X_{T}))$$

Finally, within the domain of rolling bearing fault diagnosis, the importance of marginal and conditional distributions may vary throughout the transfer procedure, with the extent of divergence between these distributions being contingent upon the specific data. Currently, most existing methods rely on grid search or random selection to determine the distribution weights, which are not only computationally inefficient but may also fail to identify the optimal solution [37]. To improve adaptability to unknown tasks, this paper proposes an adaptive factor mechanism that automatically assigns weights to the marginal and conditional distributions, thereby facilitating more effective transfer learning. The method for calculating the dynamic adaptive factor μ is presented below:

(13) $$\mu =\frac{L_{\mathrm{MDA}}}{L_{\mathrm{CDA}}+L_{\mathrm{MDA}}+\epsilon }$$

where ϵ represents a minor positive value incorporated to prevent division by zero.

In summary, the DBDA mechanism can be represented as

(14) $$L_{\mathrm{DBDA}}=(1-\mu )L_{\mathrm{CDA}}+\mu L_{\mathrm{MDA}}$$

where μ and 1−μ are the adaptive weights for the marginal and conditional distributions, respectively. The computation methods for the CDA loss and MDA loss are shown in Equations (11) and (12), respectively.

### 2.6. Loss Function of the AttenResNet18

In the AttenResNet18 model presented in this study, the loss function comprises two primary components: the DBDA loss and the classification loss. This configuration aims to concurrently reduce domain differences and improve classification precision.

Initially, the DBDA method aligns the distributions between different domains by dynamically tuning the CDA and MDA loss weights using a factor μ, as detailed in Equation (14).

Next, to boost the distinctiveness of features, the I-Softmax loss [26] is utilized as the optimization criterion for the classifier, with its definition provided below:

(15) $$L_{y}=\left\{\begin{array}{cc} -\frac{1}{n}\sum_{i=1}^{n}\log\left(\frac{e^{F^{i}(c)/m-k}}{e^{F^{i}(c)/m-k}+\sum_{j\neq c}e^{F^{i}(j)}}\right), & F^{i}(c)>0\\ -\frac{1}{n}\sum_{i=1}^{n}\log\left(\frac{e^{mF^{i}(c)-k}}{e^{mF^{i}(c)-k}+\sum_{j\neq c}e^{F^{i}(j)}}\right), & F^{i}(c)\leq 0 \end{array}\right.$$

where n denotes the overall count of feature vectors and $F^{i}$ denotes the output of the feature vector. $F^{i}(c)$ and $F^{i}(j)$ denote the *c*-th component and other components associated with the label index of $x_{i}$, in our experiments, k=16, m=3, which serve as hyperparameters controlling the decision boundary.

The overall classification loss integrates both the supervised loss derived from the source domain and the pseudo-label loss generated from the target domain.

(16) $$L_{C}=L_{S}+\gamma L_{T}$$

where $L_{S}=\frac{1}{N_{S}}\sum_{i=1}^{N_{S}}L_{y}^{\left(S\right)}(x_{i},y_{i})$, $L_{T}=\frac{1}{N_{T}}\sum_{j=1}^{N_{T}}L_{y}^{\left(T\right)}(x_{j},{\hat{y}}_{j})$, and ${\hat{y}}_{j}=\arg\max F^{l}(x_{j}^{\left(T\right)})$. Equation (15) provides the specific calculation method for $L_{y}$. The hyperparameter γ is set to 0.1, considering the difference between the pseudo-label and the true label.

In conclusion, the total loss of AttenResNet18 can be expressed as follows:

(17) $$L=L_{C}+L_{\mathrm{DBDA}}$$

## 3. Experimental Validation

### 3.1. Dataset Description

#### 3.1.1. CWRU

**Figure 5:**
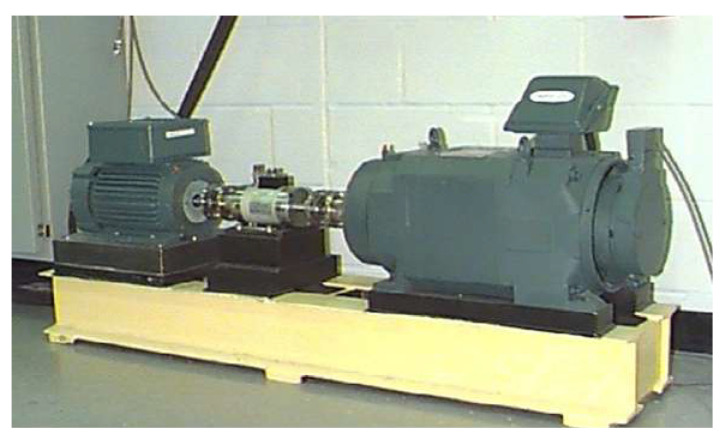
CWRU experiment test rig [38].

The Case Western Reserve University (CWRU) Bearing Fault Dataset is a widely utilized resource in bearing fault diagnosis research due to its comprehensive vibration data from bearings operating under various fault conditions and modes. The experimental setup, illustrated in Figure 5, includes a 2 hp motor, multiple test bearings, a torque transducer, a dynamometer, and associated control electronics. Accelerometers at the drive end, fan end, and base plate record vibration signals, with the motor running at four different load conditions: 0 hp at 1797 rpm, one hp at 1772 rpm, two hp at 1750 rpm, and three hp at 1730 rpm. The dataset comprises signals from healthy bearings and those with faults in the ball, inner race, or outer race, where the diameters of the defects span four sizes: 0.007, 0.014, 0.021, and 0.028 inches. Vibration data from the drive-end accelerometer, sampled at 12 kHz, are used in this study.

#### 3.1.2. The BJTU-RAO Bogie Dataset

**Figure 6:**
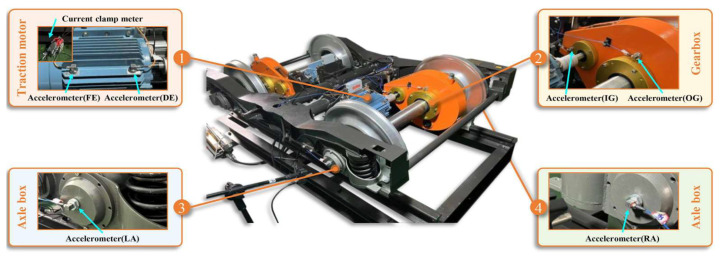
BJTU-RAO Bogie Dataset experiment test rig [39].

The BJTU-RAO Bogie Dataset is based on a reduced-scale experimental configuration designed to replicate real-world railway operations, encompassing individual and combined fault categories. As depicted in Figure 6, the test rig incorporates a power transmission system featuring traction motors, driving gearboxes, and axle boxes. During data acquisition, the experimental parameters controlled included motor speeds of 20, 40, and 60 Hz (simulating different train velocities) and lateral loads of 0, +10, and −10 kN (representing straight-line travel and curve negotiation, where positive and negative values represent loading directed toward the motor and gearbox sides, respectively). The dataset comprises four signals acquired from 24 channels, triaxial vibration, three-phase current, rotational speed, and acoustic data, each sampled at 64 kHz for 10 s. For this research, acceleration data from channel 16, situated at the gearbox output shaft, were evaluated to examine bearing faults within the gearbox.

### 3.2. Fault Diagnosis

All experiments in this study were conducted using the PyTorch 2.3.0 framework and performed on an NVIDIA GeForce RTX 3060 GPU (Nvidia Corporation, Santa Clara, CA, USA). The input signals were processed using a sliding window method with a window size of 3600 and a step interval of 600. The learning rate began at 0.0003. For model optimization, we employed the Adam optimizer alongside a StepLR learning rate scheduler, which reduced the learning step size by 1% every five epochs to support smooth convergence. The training procedure lasted for 100 epochs, using batches containing 256 samples. Detailed information regarding the datasets and experimental results is provided in the following sections. The fault category labels used in both the four-class and seven-class classification tasks are presented in Table 3.

#### 3.2.1. Case 1: Fault Transfer Diagnosis Across Different Working Conditions on the Same Machine

1. **Implementation details**

The two datasets presented in Table 4 were used to construct six transfer tasks across conditions, aimed at evaluating the model’s effectiveness under different loads and speeds: A → C, C → A, B → D, D → B, A + B → C + D, and C + D → A + B. For instance, the A + B → C + D task represents a transfer from A and B to C and D. The training set includes labeled data from A and B (source domains) and unlabeled data from C and D (target domains). In contrast, the test set contains only unlabeled samples from C and D. Every instance consists of 3600 data points, corresponding to the sliding window size. The classification tasks include two scenarios: four-class and seven-class. Their fault types and labels are shown in Table 3, where the seven-class classification additionally considers the variations in defect dimensions, specifically 0.007 inches and 0.014 inches.

2. **Experimental results and discussion**

**Figure 7:**
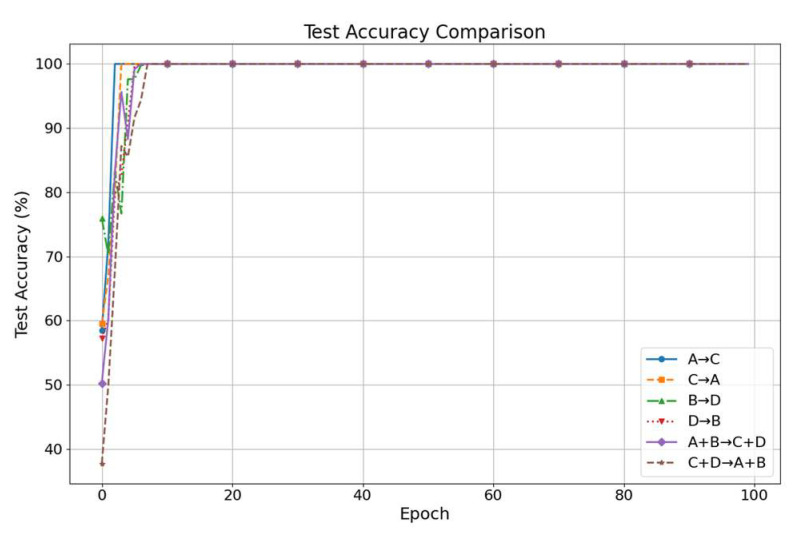
Case 1 diagnostic accuracy curves.

Figure 7 shows the accuracy trends over 100 training epochs. Accuracy increases rapidly in the first 10 epochs, reaching 100%, and remains stable thereafter. This fast convergence and sustained performance highlight the method’s efficiency and robustness.

**Figure 8:**
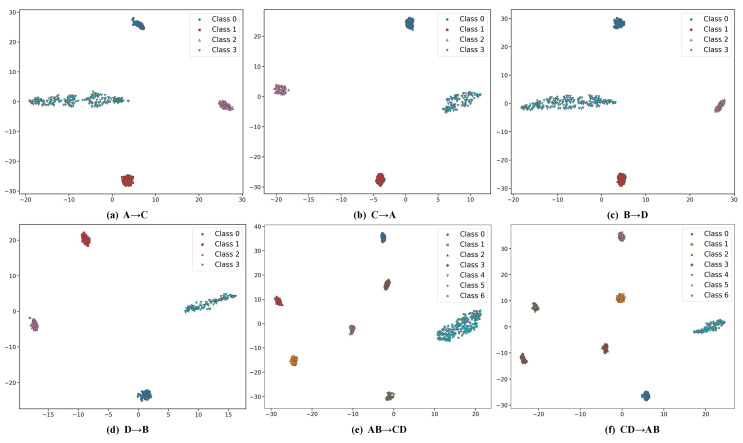
t-SNE feature visualizations for case 1.

Figure 8 illustrates the two-dimensional representations of high-dimensional data using t-distributed stochastic neighbor embedding (t-SNE) [40]. For each task, the feature groups are distinctly separated without overlap, suggesting that the model successfully captures discriminative and transferable features.

**Figure 9:**
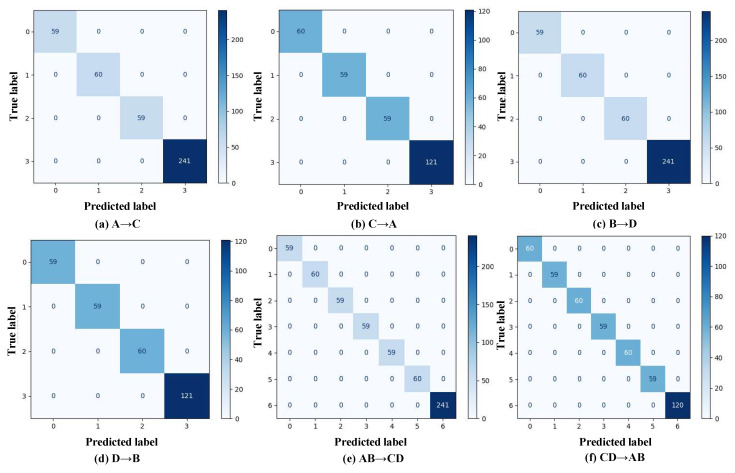
Confusion matrices for case 1.

Figure 9 displays the confusion matrices. All diagonal entries show 100% accuracy, with zero off-diagonal values, confirming perfect classification performance despite class imbalance. These results illustrate the model’s adaptability to imbalanced datasets and strong generalization ability under varying working conditions on the same machine.

#### 3.2.2. Case 2: Cross-Machine Diagnosis

1. **Implementation details**

To more thoroughly evaluate the effectiveness of the proposed method, six transfer tasks across different machines were developed: A → E, A → F, A → G, C → E, C → F and C → G. Each task is a four-class classification task, with the fault types and their corresponding labels listed in Table 3. Due to domain shifts introduced by variations in machine characteristics, operating conditions, and noise levels, cross-machine diagnosis presents increased challenges. Among all the tasks, C → E is selected for subsequent experiments for further analysis.

2. **Experimental results and discussion**

**Figure 10:**
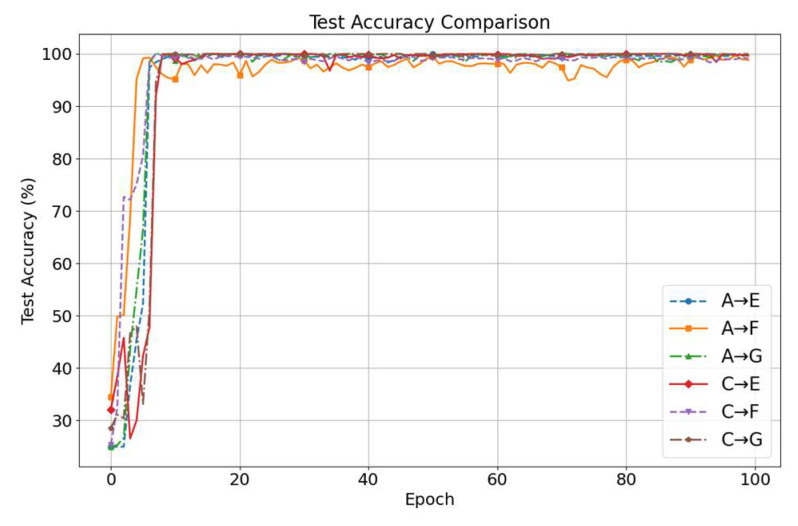
Case 2 diagnostic accuracy curves.

Figure 10 shows the diagnostic accuracy curves over 100 training epochs for the six transfer tasks. Initially, all tasks start with an accuracy of approximately 30%, rapidly increasing within the first 10 epochs and stabilizing above 95%. Final classification accuracies are as follows: A → E at 99.84%, A → F at 98.82%, A → G at 99.76%, C → E at 99.69%, C → F at 99.14% and C → G at 100%. These results demonstrate the method’s stability and generalization ability under cross-machine conditions.

**Figure 11:**
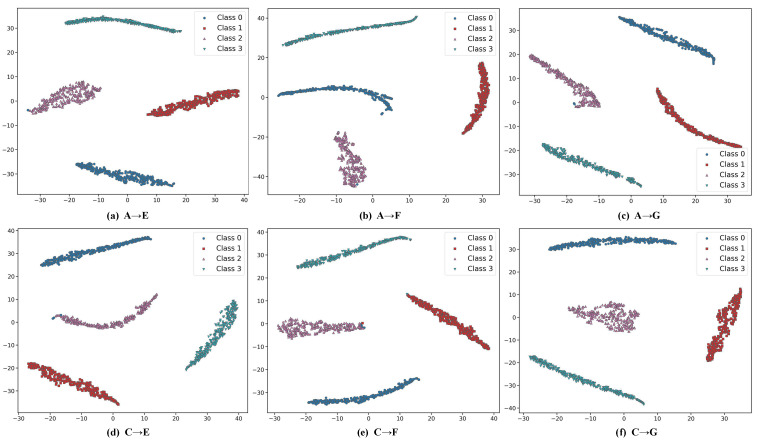
t-SNE feature visualizations for case 2.

Figure 11 shows the two-dimensional projections of features reduced by t-SNE across the six tasks. Despite occasional misclassifications (for instance, class 0 being mistaken for class 2 in the A → E and A → F tasks), the separation between classes remains distinct. These results indicate that AttenResNet18 can successfully learn discriminative and transferable features.

**Figure 12:**
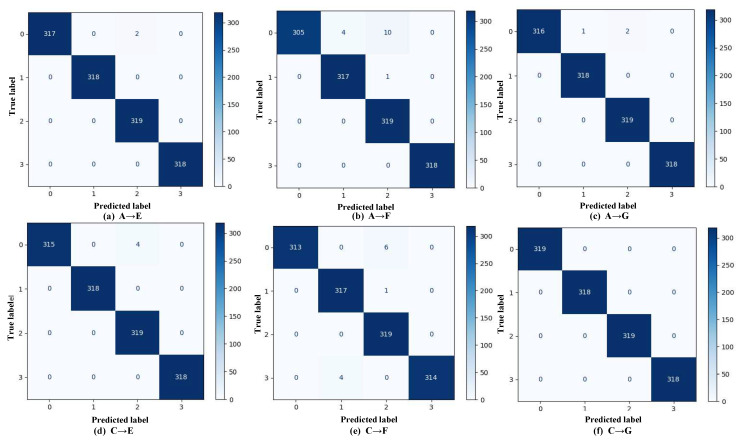
Confusion matrices for case 2.

Defects in rolling bearings can alter their multi-resonant system, thereby changing the collective behavior of elementary vibration impulses and ultimately leading to variations in the vibrational signals detected by sensor transducers [41]. Based on this principle, the model is able to perform fault classification by recognizing these vibration signals. However, the confusion matrices in Figure 12 indicate that, in case 2, two types of misclassifications still occur: false positives and inter-class errors. In the C→F task, for example, healthy bearings (class 3) are occasionally misclassified as inner race faults (class 1), representing false-positive errors. While less critical, such misjudgments can lead to unnecessary inspections or premature component replacements, thereby increasing operational costs. The most frequent inter-class misclassification occurs when ball faults (class 0) are predicted as outer race faults (class 2) and, less frequently, as inner race faults (class 1). These errors may misdirect maintenance efforts toward the outer or inner race, causing the actual ball fault to be overlooked. This can waste resources, prolong downtime, accelerate fault progression, and increase the risk of catastrophic failures. Nevertheless, the consistently high classification performance across all tasks demonstrates the proposed approach’s effectiveness in handling domain shifts among different machines.

### 3.3. Ablation Study

#### 3.3.1. Architectural Ablation: One-Dimensional Self-Attention

**Figure 13:**
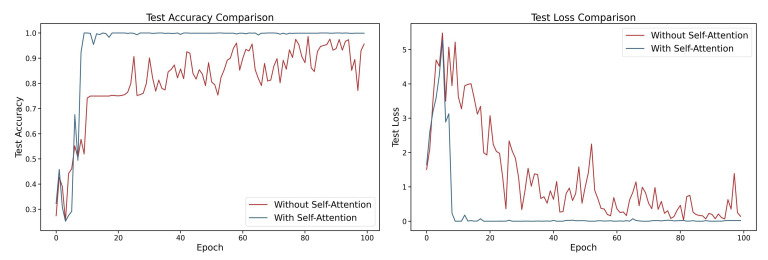
Performance comparison in self-attention ablation study: accuracy and loss curves.

To evaluate the effectiveness of the self-attention approach within the model, ablation studies were performed, with each experiment repeated five times. By comparing the performance of fault detection with the self-attention approach enabled and disabled, the mechanism’s contribution to domain-adaptive transfer learning was assessed. The results indicate that the integration of the one-dimensional self-attention mechanism led to an improvement of approximately 3.78% in test accuracy (as shown in Figure 13), demonstrating its positive impact on capturing critical features in noisy environments and enhancing the model’s cross-domain generalization capability.

#### 3.3.2. Effectiveness Analysis of DBDA

The DBDA mechanism proposed in this study dynamically allocates the weights of MDA and CDA through an adaptive factor μ, enabling flexible adaptation to different tasks. To evaluate the impact of DBDA on model performance, the Source-only model is used as the baseline. Subsequently, MMD, CORAL, MCFM, and the proposed DBDA module are introduced in sequence to construct different domain adaptation methods. Each method is tested through five independent experiments under the same dataset and experimental settings. The average classification accuracies of the methods are presented in Table 5.

**Figure 14:**
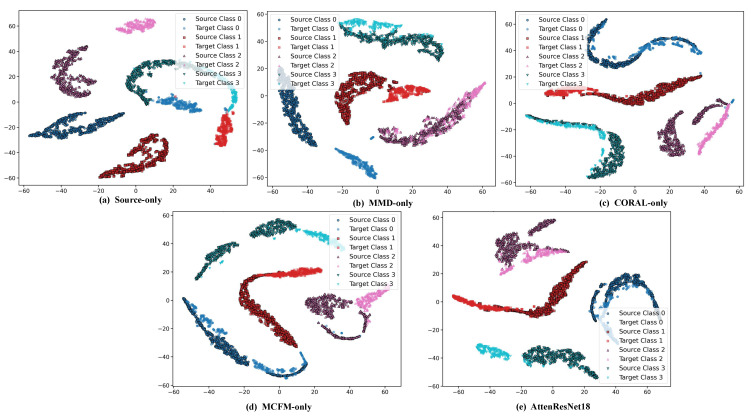
t-SNE visualization of feature distributions obtained by different methods.

As shown in Table 5, and further illustrated by the t-SNE visualization results in Figure 14, the feature distributions obtained by different methods exhibit significant differences in cross-domain tasks. For the Source-only method, the feature distributions of the source and target domains are heavily mixed, class boundaries are blurred, and samples from different categories show substantial overlap, which is consistent with its low average accuracy of 56.01%. By applying MMD, CORAL, or MCFM for global feature alignment, the feature distributions of source and target domain samples in the feature space become more similar, inter-class clustering becomes more pronounced, and overlapping regions are significantly reduced. Consequently, the average classification accuracy increases considerably to over 98%. In comparison, incorporating the proposed DBDA module into the baseline model to form the AttenResNet18 achieves the best feature separability. The source and target domain samples are highly aligned, with almost no overlap between classes, which is consistent with its highest average accuracy of 99.89% in the experiments. This strongly demonstrates its capability in cross-domain feature extraction and alignment.

#### 3.3.3. Impact of the Adaptive Factor

**Figure 15:**
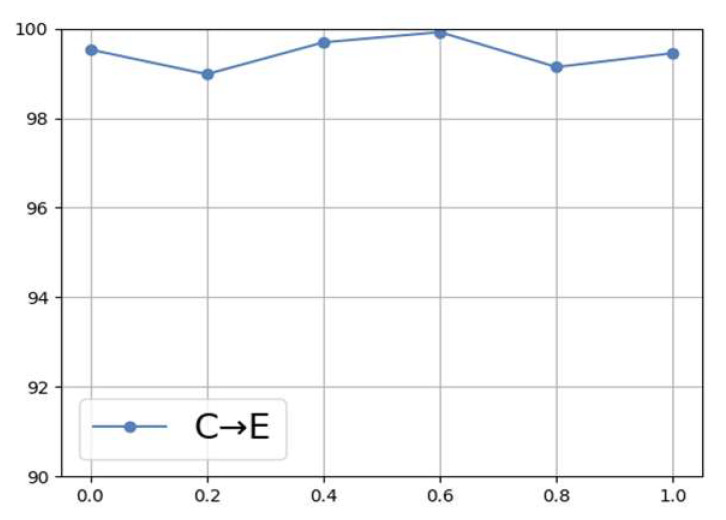
Proposed method’s diagnostic accuracy under varying adaptive factors.

To further verify the effectiveness of DBDA, Figure 15 illustrates the trend in diagnostic accuracy as the adaptive factor varies from 0 to 1. The results indicate that variations in the adaptive factor significantly influence the model’s performance. For instance, when μ=0.6, the diagnostic accuracy reaches its maximum, while deviations from this value lead to a decline in performance. This phenomenon suggests that static weight allocation is insufficient to meet the varying demands of different tasks, whereas the dynamic adjustment capability of DBDA effectively overcomes this limitation. Without the adaptive factor, one would have to manually tune the parameters to assess performance, which not only increases the workload but may also yield suboptimal results. Therefore, by leveraging the DBDA mechanism, the model can adapt more efficiently to unknown tasks, improving its reliability in practical fault diagnosis applications.

### 3.4. Noise Resistance Experiment

**Figure 16:**
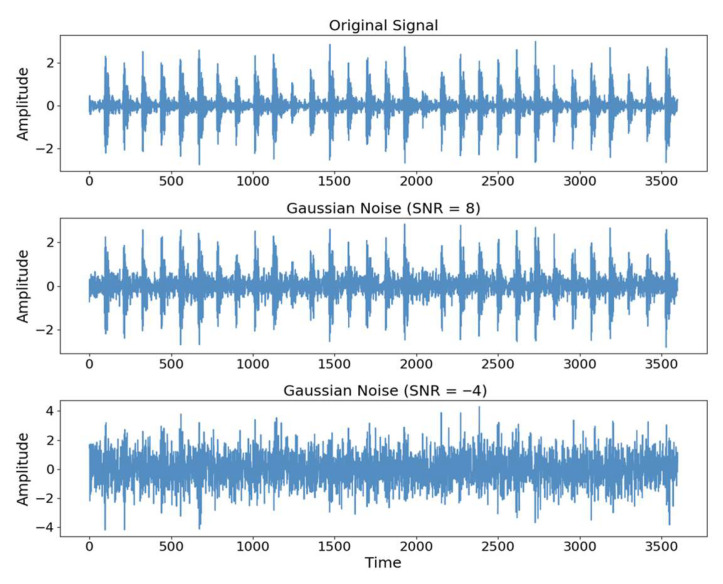
Comparison of Noise Signals under Different SNRs.

A rolling bearing vibration signal sample was randomly selected, and time-domain signal plots were obtained under various SNR conditions, as shown in Figure 16. At an SNR of 8 dB, the noise signal exhibits relatively minor deviations from the original signal. However, when the SNR drops to −4 dB, the noise component markedly obscures the characteristic features of the effective signal.

**Figure 17:**
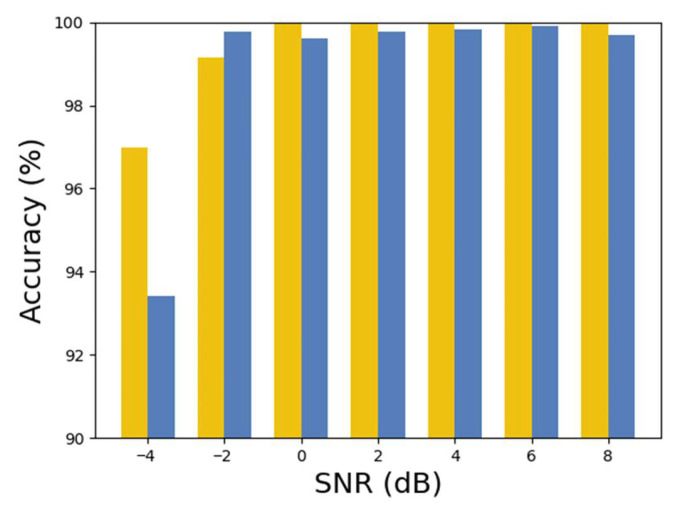
Diagnostic accuracy under various SNR levels.

With the improvement in SNR, the influence of noise on the original signal gradually weakens. To simulate the varying noise conditions encountered in real-world engineering environments, Gaussian noise with SNR, specifically from −4 dB to 8 dB, as referenced in [31], was added to the testing dataset to evaluate the model’s performance against noise. Experiments on noise resistance were conducted on the same machine for the cross-operating condition diagnostic task (A + B → C + D) and the cross-machine diagnostic task (C → E). The diagnostic results for different noise conditions are shown in Table 6 and visualized in the bar chart in Figure 17.

The accuracy rates achieved by the model were 96.98% for task A + B → C + D and 93.41% for task C → E, under an SNR of −4 dB, showcasing its effectiveness in noisy conditions. When the SNR reaches −2 dB or higher, the accuracy for both tasks surpasses 99%. These results underline the model’s superior ability to resist noise in simulated practical engineering situations across varying noise levels.

### 3.5. Comparative Experiments

To evaluate the efficacy of the introduced AttenResNet18 architecture, a comparative study was performed against several existing domain adaptation methods, including DANN [19], CDAN [21], DDTLN [26], and the Source-only baseline. To assess the robustness of our results, each method was evaluated in five independent runs, with the outcomes summarized in Table 7.

As presented in Table 7, our developed AttenResNet18 model attained a mean accuracy of 99.89%, substantially surpassing the performance of other domain adaptation methods. This outcome underscores the enhanced classification and diagnostic proficiency of AttenResNet18 relative to traditional domain adaptation techniques.

In addition to accuracy, this paper evaluates the computational efficiency of each method from three dimensions, training time, inference time, and memory usage, with the results summarized in Table 8. AttenResNet18 exhibits higher memory usage than the other models but achieves the highest accuracy. Although the per-epoch training time of AttenResNet18 is slightly longer than that of DDTLN and the source-only baseline model, its inference speed is comparable to the baseline models and significantly faster than DANN and CDAN. Furthermore, compared to the source-only model, AttenResNet18 replaces original variables with new tensors generated from intermediate computations during training, thereby releasing a portion of GPU memory in advance. This approach avoids the simultaneous residency of large-scale activation tensors on the GPU, thus significantly reducing memory consumption while attaining superior accuracy. Therefore, AttenResNet18 maintains excellent diagnostic capabilities without incurring excessively high computational costs.

**Figure 18:**
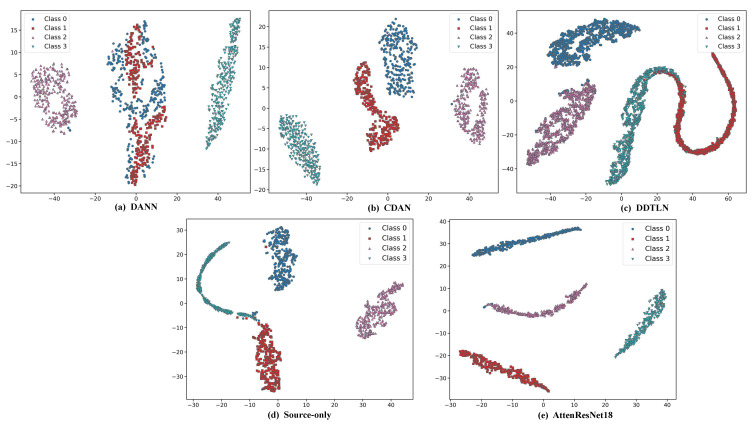
t-SNE feature visualizations for different methods.

As illustrated in Figure 18, AttenResNet18 extracts features that demonstrate the most compact within-class grouping and the most apparent between-class separation. This suggests that AttenResNet18 excels at learning features with improved class distinguishability and domain invariance, leading to enhanced performance in transfer tasks and further confirming its superiority over conventional DA methods.

### 3.6. Model Interpretability Analysis Combined with t-SNE

**Figure 19:**
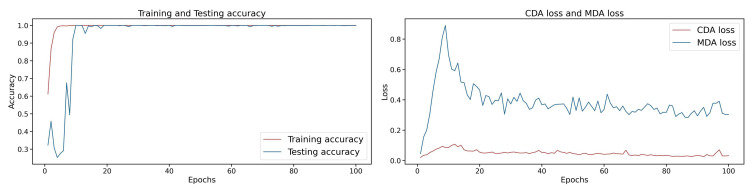
Accuracy and loss over training epochs.

**Figure 20:**
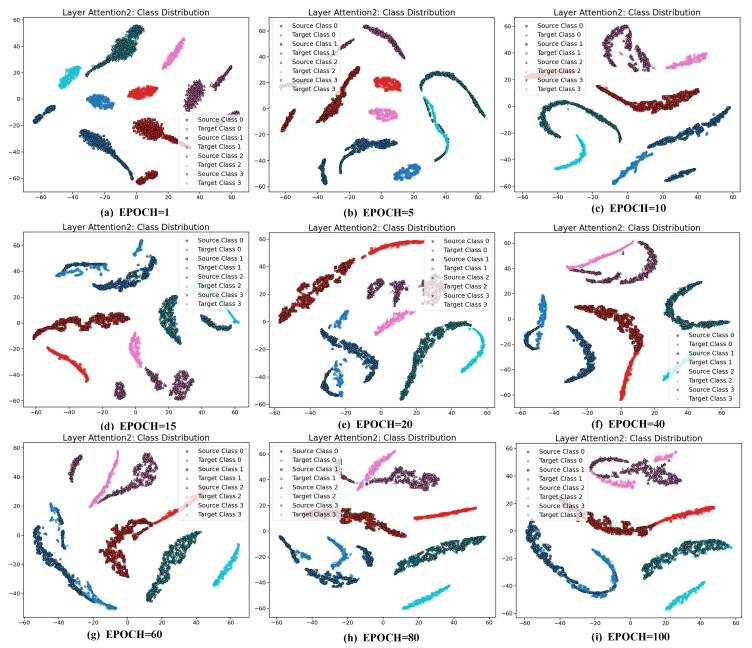
t-SNE view of source–target feature alignment.

The t-SNE visualizations can reveal how well the feature distributions between different domains are aligned. Within the AttenResNet18 network architecture (Figure 2), the Attention2 layer is positioned after the deep convolutional blocks, integrating convolutional features with self-attention optimization to produce a more semantically representative feature space. Therefore, t-SNE transforms the features derived from the Attention2 layer. As shown in Figure 19, the accuracy and MDA loss stabilize after approximately 20 epochs. Therefore, visualizations are conducted every five epochs during the first 20 epochs and every 20 epochs throughout the subsequent 80 epochs. Figure 20 displays the comprehensive visualization outcomes.

Figure 20 illustrates that during the initial phases (epochs 1 to 15), the target domain feature clusters exhibit significant differences from the source domain, with only slight overlap. This pattern reflects a typical domain shift, during which the model’s accuracy increases rapidly (Figure 19), indicating that it initially prioritizes learning from source domain features to mitigate the distribution discrepancy. During the intermediate training phases (epochs 20 to 40), the feature points progressively converged, increasing overlap and a tendency towards similar distributions. By the later stages (epochs 60 to 100), the distributions become more compact, with clear class boundaries and a significantly reduced domain gap, underscoring the effective mitigation of domain shift and the successful transfer of knowledge.

To further interpret the model behavior, we analyzed MDA and CDA losses (Figure 19). The MDA loss spikes early, likely due to noisy or unstable features. The DBDA mechanism, through dynamic adjustment of the weights of the CDA and MDA loss during training, reduces the MDA loss. After 20 epochs, both losses stabilize, matching the improved feature clustering and accuracy, confirming the method’s effectiveness.

## 4. Conclusions

Recognizing the importance of overcoming cross-domain challenges in fault detection, we introduce AttenResNet18, a model that incorporates a one-dimensional self-attention module alongside the DBDA framework. The self-attention component effectively identifies long-range feature dependencies and salient fault characteristics, reducing the impact of noise and enhancing diagnostic performance under high-noise conditions. The DBDA module leverages an adaptive weighting strategy to balance CDA and MDA, thereby improving the model’s generalization to unseen domains. Additionally, a hybrid metric, MCFM, is proposed by fusing MMD and CORAL, and embedded within DBDA to further minimize distribution divergence and improve classification accuracy. Experimental validations confirm that AttenResNet18 achieves an average accuracy of 99.89%, which is superior to existing domain adaptation techniques, highlighting its effectiveness in cross-domain bearing fault detection. This method provides a reliable diagnostic solution and contributes to the enhancement of equipment safety and operational dependability.

One noted limitation is the manual tuning requirement for data augmentation intensity, which may hinder the model’s adaptability across various fault types and noise levels, potentially affecting stability in certain scenarios. Future research could concentrate on developing methods that dynamically modify the intensity of data augmentation based on the properties of the original input signals, thus improving the model’s flexibility in intricate settings.

## Figures and Tables

**Table 1:** Techniques for data augmentation.

Technique	Parameter Range	Scope	Implementation
Gaussian noise [31]	$\begin{array}{l} {\mathrm{SNR}}_{S}\in [0,10]\\ {\mathrm{SNR}}_{T}\in [0,20] \end{array}$	Source and target domain training sets	Addition of Gaussian white noise controlled by a specified signal-to-noise ratio (SNR)
Time stretching [32]	α∈{0.9,1.1}	Source domain training set	Adjust length $L^{\prime }=\alpha L$ based on a random factor α, then interpolate to the original length (α>1: rotational speed decrease; α<1: rotational speed increase)
Time shifting [33]	s∈{−100,100}	Source domain training set	Circularly shift the signal to simulate phase changes

**Table 2:** Detailed parameters of AttenResNet18.

Layer Name			Input Size	Output Size
Conv1d_1	Convolution BN Relu	7,32,stride2	(2000×1)	(1000×32)
Pooling	MaxPool1d	3,stride2	(1000×32)	(500×32)
BasicBlock_1	Dropout = 0.35	$\left[\begin{array}{c} 3,32\\ 3,32 \end{array}\right]\times 2$	(500×32)	(500×32)
Attention1	SelfAttention1D	/	(500×32)	(500×32)
BasicBlock_2	Dropout = 0.35	$\left[\begin{array}{c} 3,64\\ 3,64 \end{array}\right]\times 2$	(500×32)	(250×64)
BasicBlock_3	Dropout = 0.35	$\left[\begin{array}{c} 3,128\\ 3,128 \end{array}\right]\times 2$	(250×64)	(125×128)
BasicBlock_4	Dropout = 0.35	$\left[\begin{array}{c} 3,32\\ 3,32 \end{array}\right]\times 2$	(125×128)	(63×256)
Attention2	SelfAttention1D	/	(63×256)	(63×256)
Classification	AdaptiveAvgPool1d Dropout = 0.5 FC	/	(63×256)	4

**Table 3:** Fault type labels for tasks.

Task	Fault Type	Label
Four-class	Ball	0
	Inner	1
	Outer	2
	Normal	3
Seven-class	Ball_007	0
	Inner_007	1
	Outer_007	2
	Ball_014	3
	Inner_014	4
	Outer_014	5
	Normal	6

**Table 4:** The datasets employed in this study.

Name	Datasets	Load	Speed	Fault Diameters
A	CWRU	0 hp	1797 rpm	0.007 in
B	CWRU	0 hp	1797 rpm	0.014 in
C	CWRU	2 hp	1750 rpm	0.007 in
D	CWRU	2 hp	1750 rpm	0.014 in
E	BJTU-RAO	0 kN	2400 rpm	/
F	BJTU-RAO	+10 kN	2400 rpm	/
G	BJTU-RAO	0 kN	3600 rpm	/

**Table 5:** Comparison of ablation experiment results.

Model	Run 1	Run 2	Run 3	Run 4	Run 5	Avg (%)
Source-only	52.98	47.57	63.03	54.63	61.85	56.01
MMD-only	98.59	99.22	98.67	98.27	98.27	98.60
CORAL-only	99.14	99.06	98.98	98.74	98.74	98.93
MCFM-only	98.51	99.06	99.14	97.80	99.14	98.73
AttenResNet18	99.69	100	99.84	99.92	100	99.89

**Table 6:** Diagnostic accuracy of the proposed model under various SNR levels.

SNR (dB)	−4	−2	0	2	4	6	8
A + B → C + D	96.98	99.16	100	100	100	100	100
C → E	93.41	99.76	99.61	99.76	99.84	99.92	99.68

**Table 7:** Experimental results.

Method	Run 1	Run 2	Run 3	Run 4	Run 5	Avg (%)
DANN [19]	59.25	47.5	51.33	51.58	67.25	55.38
CDAN [21]	72.73	77.89	73.52	68.20	70.86	72.64
DDTLN [26]	63.70	63.73	63.70	63.45	63.90	63.70
Source-only	52.98	47.57	63.03	54.63	61.85	56.01
AttenResNet18	99.69	100	99.84	99.92	100	99.89

**Table 8:** Computational efficiency comparison of different methods.

Method	Training Time (s/epoch)	Inference Time (s/epoch)	Memory Usage (MB)
DANN [19]	7.62	1.60	966.17
CDAN [21]	8.67	1.60	1118.79
DDTLN [26]	6.73	0.11	234.19
Source-only	6.40	0.25	9142.12
AttenResNet18	8.02	0.25	8302.70

## Data Availability

The data that support the findings of this study are not publicly available.

## List of Abbreviations

Abbreviation	Full Term
AF	Adaptive Filtering
AttenResNet18	Attention-Enhanced Residual Network
BDA	Balanced Distribution Adaptation
CDA	Conditional Distribution Alignment
CDAN	Conditional Domain Adversarial Network
CNNs	Convolutional Neural Networks
CORAL	CORrelation ALignment
CWRU	Case Western Reserve University
DA	Domain Adaptation
DANN	Domain-Adversarial Neural Network
DBDA	Dynamic Balance Distribution Adaptation
DBN	Deep Belief Network
DDTLN	Deep Discriminative Transfer Learning Network
DRSN	Deep Residual Shrinkage Network
ECA	Efficient Channel Attention
FD-SAE	Feature Distance Stacked Autoencoder
GAN	Generative Adversarial Networks
I-Softmax	Improved Softmax
IJDA	Improved Joint Distribution Adaptation
JAADA	Joint Attention Adversarial Domain Adaptation
JDA	Joint Distribution Adaptation
LMMD	Local Maximum Mean Discrepancy
LSTM	Long Short-Term Memory
MCFM	MMD-CORAL Fusion Metric
MDA	Marginal Distribution Alignment
MMCLE	Multi-Channel and Multi-Scale CNN-LSTM-ECA
MMD	Maximum Mean Discrepancy
RKHS	Reproducing Kernel Hilbert Space
RQA-Bayes-SVM	Recurrence Quantification Analysis–Bayesian Optimization–Support Vector Machine
SNR	Signal-to-Noise Ratio
SSA	Salp Swarm Algorithm
SVM	Support Vector Machine
t-SNE	t-distributed Stochastic Neighbor Embedding
UDA	Unsupervised Domain Adaptation
VMD	Variational Mode Decomposition
